# Buffering against Depressive Symptoms: Associations between Self-Compassion, Perceived Family Support and Age for Transgender and Nonbinary Individuals

**DOI:** 10.3390/ijerph18157938

**Published:** 2021-07-27

**Authors:** Steven Samrock, Kai Kline, Ashley K. Randall

**Affiliations:** Counseling and Counseling Psychology, Arizona State University, Tempe, AZ 85287, USA; kkline5@asu.edu (K.K.); Ashley.K.Randall@asu.edu (A.K.R.)

**Keywords:** transgender, nonbinary, depression, self-compassion, family support, age

## Abstract

Transgender and gender nonbinary (TGNB) individuals often report higher levels of depression compared to cisgender individuals. Higher levels of depression in TGNB populations may be partially attributed to a lack of family support, which may be particularly salient for younger individuals. However, two possible protective factors that may mitigate depressive symptoms are self-compassion, defined as an attitude of kindness and understanding towards one’s own imperfections, and perceived support, especially from family. The present study aimed to explore whether self-compassion was negatively associated with self-reported depressive symptoms, and whether perceived family support moderated this association, especially for younger individuals. Participants who were (1) at least 18 years of age, (2) identified as TGNB, and (3) experienced gender dysphoria were eligible for this study. Cross-sectional data from 148 individuals were collected online during May 2020. In support of the hypotheses, self-compassion was negatively associated with depressive symptoms, and perceived family support furthered this association. Additionally, results showed that younger participants (ages 18–24) with lower family support reported the highest levels of depressive symptoms. Taken together, these results suggest that self-compassion and perceived family support may be significant protective factors against depressive symptoms for TGNB individuals, although longitudinal research is needed. Taking a strengths-based perspective, mental health clinicians working with TGNB individuals may consider interventions geared toward increasing self-compassion in daily life and working with clients’ families to increase support.

## 1. Introduction

### 1.1. Depression in Transgender and Gender Nonbinary Individuals

Depression is the leading cause of disability worldwide [1]. Individuals with varying gender identities may be disproportionally affected by stress, which, if not properly dealt with, can lead to depressive symptoms. For example, individuals who identify as nonbinary, gender nonconforming, gender fluid, and/or transgender (hereafter transgender and gender nonbinary [TGNB]) experience higher rates of depression compared to cisgender individuals, with a 12-fold higher rate of suicidal thoughts and attempts [2,3]. Additionally, risks of depression increase 4-fold for members of the TGNB population that do not receive gender-affirming treatment [4]. Compared to cisgender populations, higher rates of depressive symptoms among TGNB populations have been attributed to unique stressors impacting TGNB individuals, notably experiences of discrimination, harassment, violence, and lack of social support; all considered forms of minority stress [4].

Within the United States, recent discriminatory political and legislative measures (e.g., South Carolina’s bathroom bill; and the 100-plus anti-TGNB bills that have been introduced in 33 states as of April 2021) have increased TGNB visibility; visibility that has been associated with increased levels of discrimination [5]. Thus, it is imperative that researchers and clinicians alike expand the relatively small body of literature on TGNB mental health, especially given recent social events and changes in the United States. Further, identifying protective factors that may buffer against the mental health effects of discrimination are particularly salient during this historical moment.

### 1.2. Age as a Predictor of Depression in TGNB Individuals

Individuals across the lifespan may experience disproportionate rates of depression. Notably, there is mixed evidence regarding the association between age and depression in TGNB populations. On the one hand, studies have found that younger age is associated with greater incidents of higher rates of depression [6,7,8,9,10]. Bouman et al. (2016) conducted a match-case controlled survey study and found age to be a predictor of general psychopathology across nine primary symptom dimensions, including depression [6]. Age was found to be a significant predictor of psychopathology in only transgender participants, as compared to cisgender participants [6], suggesting that younger TGNB people face particularly high levels of stress which can affect mental health. One rationale for the negative association between age and depressive symptoms is that with age comes more developed coping strategies, whereas younger individuals have less experience managing stress, which negatively impacts mental health [11]. On the other hand, Witcomb et al. (2018) conducted a matched control survey study comparing question responses from 913 transgender people matched by age and experienced gender [4]. Results showed older age significantly predicted self-report depressive symptoms. According to Witcomb et al. (2018), one possibility for this association is that that there was a cumulative effect of other variables over the years (e.g., a longer period of lacking social support); and/or a greater prevalence of transphobia in older generations—that could explain why depression was predicted by older age [4]. Taken together, while age appears to predict mental health symptoms in TGNB, it remains unclear whether older age or younger age predicts higher levels of depressive symptoms.

### 1.3. Self-Compassion as a Protective Factor

Given the high rates of depressive symptoms for TGNB individuals [4], it is important for mental health clinicians to take a strength-based approach to fostering mental well-being in their TGNB clients. A strengths-based approach allows researchers and clinicians alike to identify both individual and relational factors that may buffer symptoms of depression. For example, research has shown that self-compassion is negatively associated with depressive symptoms in TGNB adults [12,13,14,15]. According to Neff (2010), self-compassion is defined by three main elements: treating oneself in a positive way during difficult times; recognizing that all humans are imperfect; meeting negative thoughts and emotions with mindfulness [16].

Individuals who report higher self-compassion scores also report higher rates of well-being, which has been thought to be a protective factor against depressive symptoms [17,18,19,20]. Indeed, cross-sectional research has shown that higher self-compassion prospectively predicts lower depression symptoms in general populations, e.g., [17,18,19,20]. Additionally, research with general populations has found that people with high self-compassion are at a relatively minimized risk of depression when faced with negative life events [21]. Theoretically, self-compassion may minimize perceived threat, thus defusing the effects of negative life events [22,23].

Despite the well-documented association between self-compassion and psychological well-being, to our knowledge, no studies have examined the association between self-compassion and depressive symptoms in TGNB individuals. However, a limited number of studies have examined self-compassion in TGNB individuals in association with other mental health constructs, including emotional distress. For example, Keng and Liew (2016) found that self-compassion moderated the negative association between gender nonconformity and subjective well-being in a Singaporean sample 206 gender nonconforming adults [12]. In another study, Allen (2017) found that self-compassion was negatively associated with emotional distress in a sample of 234 transgender individuals [13]. More recently, Gorman et al. (2020) found in a sample of 30 transgender and gender diverse participants that self-compassion was reported as a significant and effective coping strategy for managing gender-related stress in their lives [15]. Taken together, this research suggests that self-compassion may be a naturally occurring source of resilience for TGNB individuals; one that would support psychological well-being in this population.

### 1.4. Family Support as a Protective Factor

Beyond self-compassion, perceived social support, especially from family, may protect against depression for TGNB individuals [4,24,25,26]. While discrimination from family members remains an unfortunate reality for many TGNB individuals, there is a growing number of TGNB people who report receiving support from their families [24]; support that, in turn, has been associated with lower levels of psychological distress [24]. Similarly, in a sample of 154 TGNB youths (age 13 to 21), Weinhardt et al. (2019) concluded that those with greater family support were less likely to report a mental health problem in the past year [25]. Notably, the absence of family support has been found to be positively associated with depression [26]. Taken together, research suggests that perceived family support may be negatively associated with depressive symptoms.

### 1.5. Present Study

Given the disproportionate rate at which TGNB individuals experience depressive symptoms [2,4], the present study sought to identify potential individual (i.e., self-compassion) and relational (i.e., family support) protective factors in a sample of 148 TGNB individuals, which fills a gap in the literature by exploring the potential negative association between self-compassion and depressive symptoms, more specifically. Given the existing research that suggests that self-compassion is negatively associated with depressive symptoms [12,13,14,15], it was hypothesized that self-compassion would be negatively associated with self-reported depressive symptoms (H1). Additionally, based on research to suggest that family support [6,24,25,26] and older age [6,7,8,9,10] buffer against depressive symptoms, it was hypothesized that perceived family support (H2) and age (H3) would moderate the association between self-compassion and depressive symptoms. Lastly, the study authors hypothesized that family support and age would interact to moderate the association between self-compassion and depressive symptoms (H4).

## 2. Materials and Methods

### 2.1. Recruitment

Prior to data collection, the present study was approved by Arizona State University’s Institutional Review Board (IRB; ID: STUDY00011896). Participants were recruited online via various social media platforms (e.g., Facebook, Instagram, Twitter) belonging to TGNB community activists, and online TGNB support groups via Facebook. Additionally, snowball sampling techniques were used by asking participants to share this study upon completion with their own networks.

### 2.2. Participants

Participants had to meet the following inclusion criteria: (1) over the age of 18, (2) identify as transgender or gender nonconforming, and (3) experience dysphoria, defined as the distress experienced with the incongruence between one’s physical body and gender identity.

Two hundred and twenty-five participants expressed interest in the survey. Seventy-seven participants were removed for incomplete data and/or ineligibility. The final sample included 148 TGNB participants.

In terms of gender identity, most participants self-identified as transgender man (*n* = 89, 60.1%), followed by trans masculine (*n* = 19, 12.8%), nonbinary (*n* = 16, 12.8%), transgender woman (*n* = 8, 5.4%), other/not listed (*n* = 7, 4.7%), genderqueer/genderfluid (*n* = 6, 4.1%), and agender (*n* = 3, 2.0%). The participants ranged in age from 18 to 57 years-old, with a mean age of 26.38 years old (*SD* = 7.31). Just under half of the sample (*n* = 73, 49.3%) were emerging adults, aged 18–24; 40% of the sample were young adults, aged 25–35 (*n =* 58, 39.19%), and approximately 11.5% of participants were adults, aged 36–64 (*n* =17, 11.49%). (Specific categorizations of age were determined based on the American Psychological Association’s definitions, which can be accessed here: https://dictionary.apa.org/emerging-adulthood (accessed on 17 June 2021), https://dictionary.apa.org/adulthood (accessed on 17 June 2021).)

Most participants self-identified as non-Hispanic White (*n* = 96, 64.9%), followed by other/not listed (*n* = 23, 15.5%), Latinx (*n* = 17, 11.5%), Native American (*n* = 5, 3.4%), Black (*n* = 4, 2.7%), and Asian (*n* = 3, 2%). Eighty-one participants (54.7%) reported an income of $0–24,999; forty-eight participants (32.4%) reported an income of $25,999–49,999; eighteen participants (12.2%) reported an income of $50,000 and above. Education level ranged from those who had not attended college (*n* = 32, 21.7%); to those who had attended some college (*n* = 53, 35.8%); to those with an undergraduate degree (*n* = 44, 29.7%); and a graduate degree (*n* = 19, 12.8%). See Table 1 for complete sociodemographic information.

### 2.3. Screening and Survey

Interested participants either contacted the research team via email to receive the Qualtrics survey link or clicked directly on the survey Qualtrics link that was included in online recruitment posts. The first page of the Qualtrics survey link contained the informed consent. Following completing the informed consent, there was an initial screening survey to determine eligibility (see eligibility requirements in Recruitment above). Eligible participants were directed to the study survey. The survey took approximately 20 min to complete. Upon completion, participants were able to opt into a raffle for a chance of one of nine $25 Amazon e-gift cards.

### 2.4. Measures

#### 2.4.1. Demographics

Participants answered standard demographic items (e.g., age, race, gender identity, income, and education).

#### 2.4.2. Self-Compassion

Self-compassion was assessed using the Self-Compassion Scale-Short Form (SCS–SF; [27]). Participants rated 12 items on a five-point Likert scale in which 1 = *Almost Never* and 5 = *Almost Always*. An example SCS-SF items is, “When I’m going through a very hard time, I give myself the caring and tenderness I need.” To calculate the total self-compassion score, six negative subscale items were reverse coded before computing a total mean. Based on the study sample, the reliability was high (Cronbach’s α = 0.92).

#### 2.4.3. Depressive Symptoms

Depressive symptoms were assessed utilizing the Center for Epidemiological Studies Depression Scale (CES-D; [28]). Participants responded to the 20 items using 0 = *Rarely or none of the time* (less than 1 day), 1 = *Some or a little of the time* (1–2 days), 2 = *Occasionally or a moderate amount of time* (3–4 days), 3 = *Most or all of the time* (5–7 days). An example item from the CES-D is, “I felt that I could not shake off the blues even with help from my family or friends.” Total scores were calculated by summing the responses across all items. Based on the study sample, the reliability was high (Cronbach’s α = 0.92).

#### 2.4.4. Perceived Family Support

Perceived family Support was measured using a four-item subscale from the 12-item Multidimensional Scale of Social Support (MSPSS; [29]). Participants were given seven response options in which, 1 = *Very Strongly Disagree*, 2 = *Strongly Disagree*, 3 = *Mildly Disagree*, 4 = *Neutral*, 5 = *Agree*, 6 = *Strongly Agree*, and 7 = *Very Strongly Agree*. An example item from the family support subscale is, “I get the emotional help and support I need from my family.” The family support score was a mean score of the four subscale item responses. Based on the study sample, the reliability was high (Cronbach’s α = 0.94).

#### 2.4.5. Control Variables: Income, Education, and Race

Including control variables in the model is important to eliminate the influence of additional (third) variables on the associations between the variables, as measured by a regression coefficient [30].

Given the documented negative association between social factors (e.g., income and education) and depression [31,32], both income and education were controlled for in the analyses. Income was measured by asking participants to report their typical yearly individual income before taxes. Participants were given six response options namely 1 *= $0–$24,999,* 2 *= $25,000–$49,000,* 3 *= $50,000–$74,999,* 4 *= $75,000–$99,999,* 5 *= $100,000–$149,999,* and 6 *= $150,000 or more*. Education was measured by asking participants to report their highest level of education completed. Participants were given six response options namely 1 *= Less than high school,* 2 *= High school,* 3 *= Professional program,* 4 *= Some college,* 5 *= Undergraduate degree,* and 6 *= Graduate degree.* Additionally, research has shown that there are significant racial/ethnic differences in rates of depression among general adult populations [33]. Given these associations, race was added as a control variable in the regression analysis. Race was measured by asking participants report their racial background. Participants were given seven response options namely 1 *= Asian American,* 2 *= Black/African American,* 3 *= Hispanic/LatinX,* 4 *= Native American/American Indian,* 5 *= Native Hawaiian/Pacific Islander,* 6 *= Non-Hispanic White,* and 7 *= Multiracial/Other.*

None of the control variables yielded statistically significant coefficients in the regression model (i.e., income (β = 0.02, *p* > 0.05), education (β = −0.07, *p* > 0.05), and race (β = 0.01, *p* > 0.05). Given this, the control variables were removed from the model for parsimony.

### 2.5. Data Analysis

Prior to hypothesis testing, the data were checked for normality. Skewness and kurtosis variables fell within the acceptable range for the study variables (self-compassion: skewness = −0.75, kurtosis = 1.26; depression: skewness = 0.12, kurtosis = −0.29; family support: skewness = 0.04, kurtosis = −0.96).

Prior to hypotheses testing, correlational analyses were conducted using SPSS version 26 [34], the researchers first conducted bivariate Pearson correlations to test the strength of correlations among study variables Taking the absolute value of the correlation coefficient allowed us to classify the associations as small, moderate, and large if the values were above 0.10, 0.30, and 0.50, respectively [35].

To test our hypotheses, data were analyzed using moderation models via the Hayes’ PROCESS macro version 3 (Hayes, 2013) for SPSS. In the moderation model, the independent variable was the standardized score for self-compassion, and the dependent variable was the standardized score for depressive symptoms. The moderator variables were the standardized score for perceived family support and age. Additionally, there were three covariates added: standardized income, education, and race. In PROCESS version 3, percentile bootstrap confidence intervals are the default, meaning that the confidence intervals are determined by the random resampling bootstrapping method. In this model, the number of bootstrap samples were set to 5000 to minimize sampling error in the estimation of the end points of the confidence interval.

## 3. Results

Results showed significant negative associations between depressive symptoms and self-compassion (*r =* −0.27, *p* < 0.01), family support (*r =* −0.24, *p* < 0.01), and age (*r =* −0.26, *p* < 0.01) These values reflect small effects. These results suggest that lower scores in depressive symptoms were associated with higher scores in self-compassion, perceived family support, and older age. It is important to note that the values of the correlation terms were low, which reflects weak associations among variables, which may be reflective of the smaller sample size. See Table 2.

First, consistent with H1, results showed that there was a significant main effect of self-compassion on depressive symptoms, β = −0.21, *p* = 0.01, such that higher reported levels of self-compassion were associated with lower levels of reported depressive symptoms. See Table 3.

Second, contrary to H2, perceived family support did not moderate the association between self-compassion and depressive symptoms, (β = −0.19, *p* > 0.01), nor did age (β = −0.10, *p* > 0.01). Self-compassion and family support did not interact to moderate the association. Nor was there a significant interaction between self-compassion and age on depressive symptoms, β = 0.16, *p* > 0.01.

**Figure 1:**
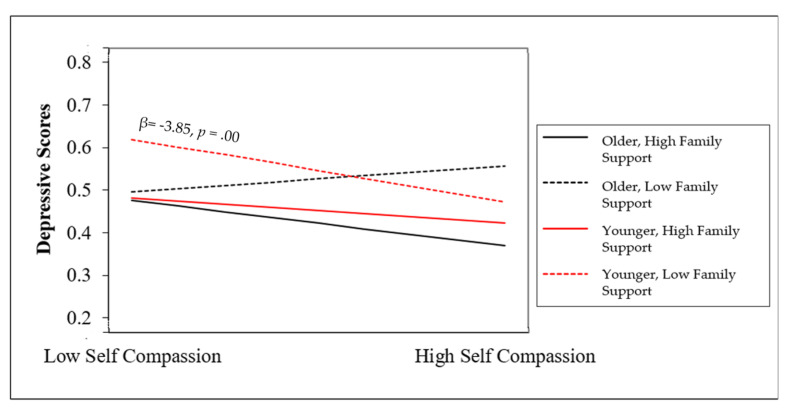
Interaction between self-compassion, family support, and age on depressive symptoms.

Lastly, with regard to H4, there was an interaction between perceived family support and age on the association between self-compassion and depressive symptoms. β = −0.26, *p* < 0.01. The three-way interaction predicted a significant portion of variance in depressive symptoms beyond self-compassion (F = 4.93, *p* < 0.01). Based on the simple slope analysis [36], younger participants (ages 18–25) with lower family support showed the highest levels of depressive symptoms (β = −3.85, *p* < 0.01). This indicates that younger participants with low self-compassion and low perceived family support reported the highest levels of depressive symptoms. See Figure 1.

A Bonferroni correction was applied to the findings to control the overall α-level.

## 4. Discussion

TGNB individuals experience higher rates of depression compared to cisgender individuals [2,4]. As such, it is important to identify possible protective factors for TGNB individuals, especially in light of the recent sociopolitical climate in the United States, where new waves of legislation are being introduced to limit TGNB people’s rights. While the body of literature of protective factors for TGNB individuals has grown in recent years, e.g., [4,13,14,15,24,25,26], further examination of specific protective factors is needed. Based on a sample of 148 TGNB individuals living in the United States, results showed higher reported self-compassion was associated with lower reported depressive symptoms. This association was moderated by perceived familial support and age, such that higher levels of self-compassion and family support were associated with the lowest levels of depressive symptoms.

### 4.1. Self-Compassion and Depressive Symptoms

Self-compassion—the positive ways in which one treats oneself during difficult times—is considered an effective coping strategy for TGNB individuals [15]. The results of the present study showed that self-compassion in TGNB individuals was negatively associated with depressive symptoms, which supports previous research [13,14,15]. While this study used cross-sectional data and cannot establish temporal associations between self-compassion and depression (see Limitations), theorists have postulated that self-compassion is a protective factor against symptoms of depression [22,23]. This study’s results supplement those findings, specifically highlighting the potential importance of self-compassion for TGNB individuals.

### 4.2. Moderating Effects of Perceived Family Support and Age on Depressive Symptoms

In line with existing research that demonstrates practicing self-compassion is associated with better mental health for TGNB individuals [13,14,15], the present study found self-compassion to be negatively associated with depressive symptoms. Additionally, this association was moderated by perceived family support and age, which supports prior research that has found perceived family support to be an important protective factor for TGNB individuals’ level of depressive symptoms [4,24,25,26]. Particularly in younger participants with low perceived family support, self-compassion buffered against depressive symptoms. The results of this study show that, while family support and self-compassion interacted to buffer depressive symptoms, this interaction was further contextualized by age.

### 4.3. Limitations

While this study adds to the understanding of protective factors that may buffer against depressive symptoms in TGNB individuals, it is not without limitations. The smaller sample size (G*Power 3.1 [37] was utilized to calculate the total sample size needed for the current study (*n* = 146)) and lack of variability in participant demographics limit the generalizability of the study findings. Specifically, more than half of the study participants self-identified as transgender men and trans masculine. The lack of representation of transgender women and trans feminine participants makes it difficult to extend the study findings to these populations. Additionally, generalizability to TGNB individuals across the life course is limited due to a sample that skews younger. Because just under half of the sample were emerging adults (aged 18–24), just under 40% of the sample were young adults (aged 25–35), and only 11.5% of participants were in middle adulthood (aged 36–64), the results and conclusions may be more applicable to the younger participants. Perhaps related to the small sample size as well, the values of the association terms were low, indicating that the associations are weak and other factors are important contributors to mental well-being in this population.

Given identified gender differences related to the present study variables (e.g., self-compassion [38] and depression [39]), it is possible that gender differences would be present in the current study if a more diverse study sample was collected. Specifically, previous research has identified higher reports of depressive symptoms in women than men, with a lack of research examining differences in gender expansive identities [39]. Similarly, existing research has highlighted higher levels of self-compassion in males than females [38]. The described research focuses on binary categorization of gender identity and does not address gender expansive individuals, further highlighting the need for exploration of study variables with gender expansive individuals.

Furthermore, the present study sample primarily self-identifies as non-Hispanic White, limiting the extent to which results may apply to TGNB individuals of color. In line with intersectionality theory [40], one’s social position is multi-dimensional; societal systems of gender and race can impose multiple oppressions, thus creating a more complex and challenging social experience in which TGNB individuals of color must overcome many barriers such as transphobia and racism [41,42]. Understanding these intersections, especially for TGNB individuals, remains an important area for future research.

Another limitation is related to the study design and measurement. Specifically, inherent to the methodology of cross-sectional data collection, the results cannot demonstrate causality between self-compassion and depression symptoms; nor examine the temporal associations between perceived (and received) support and depressive symptoms. Related to the study’s measurement, there are likely limitations with respect to additional covariates that may have impacted reports of depressive symptoms, such as suicide attempts or drug use. Lastly, the scales utilized in this study were developed and validated in cisgender populations, potentially leading to measurement error and limited validity [43]. The need for empirically validated self-report measures for use with TGNB individuals is important in future research.

Lastly, it is important to take into consideration when these data were collected. The study’s data were collected in May of 2020, two months after the World Health Organization declared COVID-19 a pandemic [44]. The COVID-19 pandemic has brought psychological distress upon many populations around the globe, and young people and TGNB individuals, in particular, have faced unprecedented difficulties with mental health amidst the pandemic [45,46]. Due to hospitals and medical facilities being overwhelmed, TGNB individuals reported facing increased barriers to health care, hormone interventions, and gender-affirming surgeries [47]. These struggles, compounded with existing (minority) stressors, were associated with high reported rates of psychological distress. Thus, the context of when the data were collected should be considered when interpreting the results of the present study, such as the potential for higher baseline levels of depressive symptoms as compared to pre-pandemic, although these data are not available.

### 4.4. Future Directions

Apart from the notable areas for future research described above, additional research that lends itself to important clinical and policy recommendations for TGNB individuals is needed. Specifically, further research is needed to understand how TGNB individuals develop and implement protective factors in their daily lives, and how these factors may change over time. At a basic level, researchers are encouraged to develop and/or test existing measures that can be used reliably with TGNB individuals. One fruitful area for research would be to better establish and understand causality between self-compassion, perceived family support, and depression by testing longitudinal clinical outcomes in intervention studies with TGNB samples. Importantly, given holding multiple marginalized identities can compound and amplify discriminatory experiences [39,40,41], more intersectional research (e.g., research with young TGNB people of Color) is needed to understand how protective factors may differ for TGNB individuals with multiple marginalized identities.

### 4.5. Clinical Implications

The results of the present study have important implications for psychologists and other mental health professionals (hereafter clinicians) working with TGNB clients. First, it is important for clinicians to understand the negative association between self-compassion and depressive symptoms, as this could be considered a foundation for clinicians to use a strength-based approach to strengthen protective factors for TGNB clients. Along these lines, Neff and Germer (2012) developed an eight-week group intervention designed to teach people to become more self-compassionate in daily life [48]. The intervention, called Mindfulness Self-Compassion (MSC), involves interpersonal exercises, guided meditations, and informal practices (e.g., restating memorized self-compassionate phrases). While the self-compassion intervention was shown to be effective in improving resilience and well-being in female college students, there may be opportunities to adapt such brief self-compassion interventions to become more culturally relevant for TGNB populations, given their increased risk of depression [4].

Additionally, clinicians are encouraged to further recognize the importance of perceived (family) support for well-being. Understanding this information can be used to facilitate exploration of family dynamics within individual therapy sessions and incorporate the family in family therapy to help increase perceived support [49]. Indeed, evidence shows that social support interventions can be useful [50]. These interventions may be group or individual; professionally led or peer-provided; targeted toward increasing perceived support or building social skills to facilitate greater support. As such, clinicians may wish to explore their options for applying and adapting social support interventions to address TGNB clients’ needs for familial support.

Lastly, taking the present study’s results into consideration, clinicians can also enhance their work with TGNB individuals by understanding the interaction between self-compassion, perceived family support, and age on depressive symptoms. The present study indicates that perceived family support is especially salient for younger TGNB individuals; and when younger TGNB individuals lack family support, self-compassion may be especially helpful in buffering against depressive symptoms. Clinicians may use these findings as a foundation to navigate treatment and treatment goals. While each client will require an individualized treatment approach, the current study can provide clinicians with a starting point to begin exploration and intervention development with TGNB individuals presenting with symptoms of depression.

## 5. Conclusions

High self-compassion and perceived family support were both significantly associated with lower levels of depressive symptoms in TGNB individuals. Clinicians working with TGNB clients are encouraged to explore intrapersonal-level strength-based interventions, such as those focused on increasing self-compassion, as well as interpersonal family system-level interventions, such as those focused on increasing family support, to buffer against depressive symptoms. Considering these findings, the authors encourage additional research with TGNB individuals to identify and understand risk and protective factors, and intervention strategies, to support mental well-being in the transgender and nonbinary community. 

## Figures and Tables

**Table 1:** Sociodemographic Characteristics of Participants (*n* = 148).

Demographic	*n*	%
Age		
18–24	73	49.32
25–35	58	39.19
36–64	17	11.49
Race		
Asian American	3	2.03
Black	4	2.70
Latinx	17	11.49
Native American	5	3.38
Non-Hispanic	96	64.86
White		
Other	23	15.54
Gender Identity		
Trans man	89	60.14
Trans masculine	19	12.84
Nonbinary	16	10.81
Trans women	8	5.41
Other	7	4.73
Genderqueer/Gender fluid	6	4.05
Agender	3	2.03
Income ($) *		
0–24,999	81	54.73
25,000–49,999	48	32.43
50,000–74,999	14	9.46
75,000–99,999	4	2.70
100,000–149,999	1	0.68
150,000+	1	0.68
Education		
<High school	1	0.68
High school	30	20.27
Professional program	1	0.68
Some college	53	35.81
Undergraduate degree	44	29.73
Graduate degree	19	12.84

* One participant did not provide data.

**Table 2:** Correlation among Study Variables.

	*M*	*SD*	1	2	3	4
1. Depression	48.78	12.38	-	−0.27 **	−0.24 **	−0.26 **
2. Self-Compassion	29.29	11.30		-	0.22 **	0.16
3. Family Support	3.67	1.73			-	0.14
4. Age	25.91	7.31				-

**. Correlation is significant at the 0.01 level (2-tailed). M = mean; SD = standard deviation.

**Table 3:** Regression results with depressive symptoms as the outcome.

Effect	Estimate	*SE*	95% CI		*p*
Fixed Effects			LL	UL	
Intercept	−0.01	0.18	−0.36	0.35	0.98
Self-Compassion	−0.21	0.08	−0.36	−0.05	0.01
Perceived Family Support	−0.19	0.08	−0.34	−0.03	0.02
Age	−0.10	0.10	−0.29	0.10	0.33
Self-Comp × Fam Sup	0.09	0.07	−0.05	0.22	0.20
Self-Comp × Age	0.16	0.10	−0.04	0.37	0.11
Self-Comp × Age × Fam Sup	−0.26	0.08	−0.41	−0.11	0.00
Model Summary		ANOVA			
R^2^	Adjusted R^2^	F-Statistic		F-statistic Significance	
0.22	0.17	4.93		0.00	

*Note.* SE = standard error; CI = confidence interval.

## Data Availability

Materials and data can be obtained by emailing the first author at Ssamrock@asu.edu.
